# Focal fatty infiltration of the lumbar intervertebral disc: a case report and literature review

**DOI:** 10.1093/omcr/omaf027

**Published:** 2025-05-28

**Authors:** Shritik Devkota, Hritika Rathi, Samiksha Lamichhane

**Affiliations:** Department of Radiodiagnosis & Imaging, Postgraduate Institute of Medical Education and Research, Sector-12, Chandigarh, 160012, India; Department of Radiodiagnosis & Imaging, Anil Baghi Hospital, Punjab, 152002, India; Department of Radiodiagnosis & Imaging, Postgraduate Institute of Medical Education and Research, Sector-12, Chandigarh, 160012, India; Department of Radiodiagnosis & Imaging, B. P. Koirala Institute of Health Sciences, Dharan, 56700, Nepal

**Keywords:** fatty disc degeneration, magnetic resonance imaging, fatty intervertebral disc

## Abstract

An intervertebral disc is a pliable, cushioning structure present between the vertebrae in the spine. It functions as a shock absorber, minimizing friction and facilitating movement between the spinal bones. Degenerative changes are viewed as responses to injuries, whether mechanical or metabolic, rather than as distinct diseases. Degenerative disc disease is a frequent etiology of low back pain. Magnetic resonance imaging (MRI) commonly demonstrates disc desiccation, degeneration, herniation or protrusion, osteophyte formation, and facet joint arthropathy. In this case report we have specifically addressed a component of degenerative disc disease which is seen as an uncommon MRI finding of focal intradiscal fat. Although the direct impact of focal intradiscal fat on patient outcomes is not fully understood, it typically indicates advanced disc degeneration. Recognizing this finding can assist clinicians in diagnosing the extent of disc degeneration and developing appropriate treatment plans for patients with low back pain or related symptoms.

## Introduction

Back pain, a significant public health concern, is frequently associated with intervertebral disc degeneration [1]. Disc degeneration, a progressive process characterized by the breakdown of spinal components, can manifest as decreased disc height, herniation, osteophyte formation, and facet joint arthritis. On magnetic resonance imaging (MRI), normal discs exhibit T1-isointensity and T2-hyperintensity. However, in degenerative discs, T2-hyperintensity is often diminished due to desiccation. T1-hyperintensity within the disc is uncommon and can be attributed to various factors, including fat accumulation [2–4]. Calcification, hemorrhage, mucin, fat, or melanin within the intervertebral disc can cause T1-hyperintensity. Intralesional fat within the intervertebral disc is an exceptionally rare occurrence, with limited case reports in the literature. Such fat can originate either from within the disc itself or from the fatty marrow of adjacent vertebrae [3, 5]. We present a unique case of focal fatty degeneration/infiltration involving the L4-L5 intervertebral disc in a 49-year-old female.

## Case report

A 49-year-old female presented to the outpatient clinic with low back pain radiating to her bilateral lower limb, predominantly at L4-L5 dermatomal level. No history of injury, fever, or other systemic conditions was reported. Vital signs were within normal limits, but there was a positive straight leg test. On motor examination of both lower limbs, muscle strength was graded 5/5, reflexes were intact, and there was no evidence of spasticity or increased tone. The laboratory investigations & electrolytes (Table 1) did not indicate any abnormal parameters that could account for the underlying cause of the low back pain.

**Table 1:** Laboratory investigations for low back pain.

Test	Result	Normal Range	Remarks
Serum Calcium	9.5	8.5–10.2 mg/dl	Within normal limits
Serum Phosphorus	3.4	2.5–4.5 mg/dl	Within normal limits
Vitamin D (25-hydroxyvitamin D)	32	30–50 ng/ml	Within normal limits
Intact Parathyroid Hormone (iPTH)	45	10–65 pg/ml	Normal level
Complete Blood Count (CBC)	Normal	Normal range	No evidence of infection or anemia
Liver Function Tests (LFTs)	Normal	Normal range	No hepatic involvement
Renal Function Tests (RFTs)	Normal	Normal range	No renal impairment
Sodium (Na^+^)	138	135-145 mEq/l	Within normal range
Potassium (K^+^)	3.9	3.5–5.0 mEq/l	Within normal range

**Figure 1:**
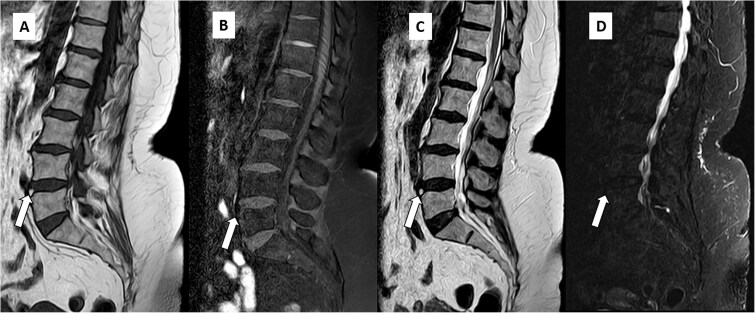
Sagittal T1W (A), T1FS (B), T2W (C), and T2FS (D) images of the patient revealing focal T1 and T2 hyperintensity in anterior aspect of the L4-L5 disc showing loss of signal and hypointensity in corresponding fat-saturated sequences suggesting fatty degeneration (arrows).

**Figure 2:**
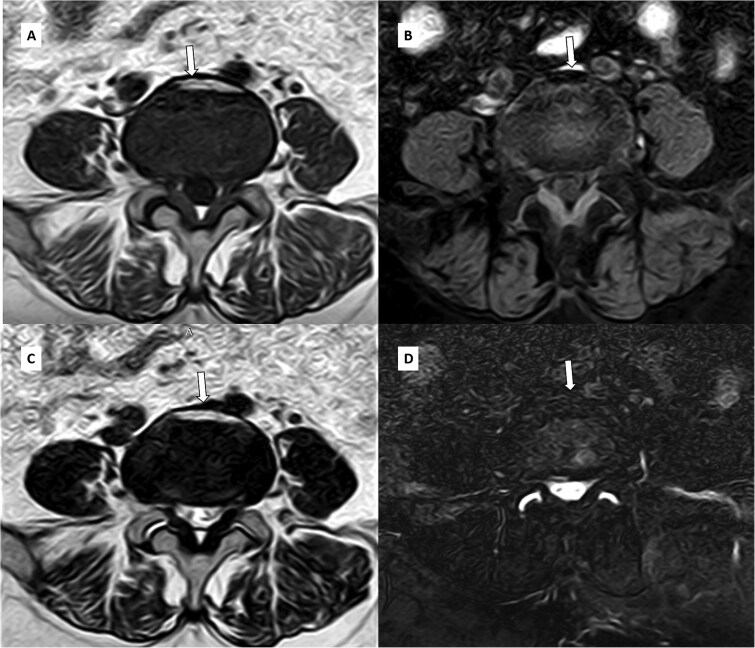
Axial T1W (A), T1FS (B), T2W (C) and T2FS (D) images at L4-L5 level revealing disc bulge at L4-L5 causing spinal canal stenosis and bilateral neural foramen compromise with focal T1 and T2 hyperintensity in anterior aspect of the L4-L5 disc showing loss of signal and hypointensity in corresponding fat-saturated sequences suggesting fatty degeneration (arrows).

Magnetic resonance imaging of the lumbar spine revealed degenerative changes, including multilevel disc desiccation and a diffuse symmetric disc bulge at L4-L5 causing minimal spinal canal narrowing and mild bilateral neural foraminal narrowing with mild compression of bilateral exiting nerve root. An unusual finding of focal T1 and T2 hyperintensity was observed on the anterior aspect of the L4-L5 intervertebral disc, which appeared hypointense on fat-suppressed sequences (Fig. 1 and Fig. 2), suggesting focal fatty degeneration/infiltration within the disc.

The patient was given analgesics, pregabalin, and methylcobalamin, along with a 3-week physiotherapy regimen. Back strengthening exercises were also advised as part of the treatment plan. A follow-up appointment was scheduled for 6 weeks, but due to lack of significant improvement, the same treatment was extended for another 6 weeks. At the 3-month follow-up, the patient reported minimal symptom relief. Consequently, surgical treatment was recommended, but the patient chose not to proceed with this option.

## Discussion

When standing upright, the spine serves as the body’s central support, akin to a mast, and fulfills three primary functions: providing structural support, allowing trunk movement, and protecting the neural components [2]. Biomechanically, the spine is a complex, multi-joint system made up of multiple segments or units which facilitates multifaceted movement and has the capacity to withstand complex forces. Each functional unit of spine consists of two nearby vertebrae, a disc between them, spinal ligaments, and the joints that connect the vertebrae [2].

The intervertebral disc, which is made of fibrocartilage, is comprised of three primary parts: the outer and the inner annulus fibrosus, and the central nucleus pulposus [2, 4]. The outer annulus fibrosus consists of densely packed, robust collagen fibers, while the inner part of the annulus fibrosus is less dense and has a mix of collagen fibers and a collagen-rich matrix [2]. The nucleus pulposus is a gel-like, semi-fluid substance. In a healthy intervertebral disc, it appears isointense on T1 and hyperintense on T2 [2, 4, 6]. Biochemical changes include decreased proteoglycans content which diminishes its capacity to retain water and preserve its gel-like texture [2–4]. The balance of collagen changes, with increased type I collagen and decreased type II collagen, which reduces the disc’s flexibility and elasticity. Fatty tissue can accumulate within the disc, a sign of advanced degeneration, which can be identified through imaging studies [2, 4, 6].

Morphological changes associated with disc degeneration include disc desiccation, a process characterized by the loss of water content, resulting in disc shrinkage and decreased flexibility. Additionally, disc height can decrease, and the outer annulus fibrosus may become more fibrous with potential tears or fissures. These structural alterations can contribute to disc bulging or herniation [2, 6].

An accurate diagnosis of a degenerative disc disease relies on a thorough medical history, an in-depth clinical examination, and advanced imaging methods such as MRI. Normally, the intervertebral disc is isointense on T1-weighted images (T1W) and hyperintense on T2-weighted images (T2W) [2, 4, 5, 7]. In degenerating disc, as the water content gradually diminishes, the disc appears hypointense on T2 [2, 3]. However, some discs may appear hyperintense on T1W images due to underlying bone marrow conditions like hematopoiesis, iron deposition, fibrosis, or tumors. These changes in bone marrow signal can cause a reversal of the typical disc/marrow signal on T1W images [3, 6–10]. The disc appears brighter than the marrow, which is called the ‘disc reversal sign’ [3]. The presence of calcification, hemorrhage, mucin, fat, or melanin within the intervertebral disc can cause T1 hyperintensity. Other causes of T1 hyperintensity within the disc include discal calcification due to unmineralized calcium. This can occur in conditions such as disc degeneration, ochronosis, ankylosing spondylitis, chondrocalcinosis, hemochromatosis, and post-operative or post-traumatic states [3, 8]. While intervertebral disc hyperintensity on both T1 and T2-weighted sequences due to fat is uncommon, a few case reports have been documented.

Similar to our case, Choudhary et al. [3] described a case of a 35-year-old female who underwent an MRI due to neck stiffness. The MRI revealed T1 hyperintensity with suppression of signal on fat-saturated T1-weighted images in the cervical intervertebral disc, suggesting fatty degeneration/infiltration.

One hypothesis suggests that tears in the annulus fibrosus, caused by trauma or degeneration, may allow epidural fat to enter the disc, particularly in the lumbar region where anterior epidural fat is present [3, 10]. Another potential mechanism involves metaplastic changes within the disc. Fatty degeneration of the intervertebral disc is generally considered a benign condition that does not require immediate intervention and can be monitored with imaging [3, 5, 7–10].

Treatment for disc degenerative disease including fatty degeneration of the intervertebral disc typically involves a combination of strategies to address symptoms and improve function. Conservative measures include physical therapy to build spinal strength and flexibility, medications such as NSAIDs to manage pain and inflammation, and lifestyle modifications like weight management and ergonomic improvements. For more advanced cases, invasive treatments such as epidural steroid injections can help reduce inflammation, and disc decompression procedures can relieve pressure on the disc. If conservative methods are ineffective, surgical options such as discectomy to remove damaged disc material or spinal fusion to stabilize the spine may be explored. Treatment plans should be customized to the individual’s specific condition and overall health, with recommendations from a healthcare provider [6].

In summary, fatty degeneration/infiltration of the intervertebral disc is a relatively rare condition marked by the accumulation of fat within the disc. Although the direct impact of focal intradiscal fat on patient outcomes is not fully understood, it typically indicates advanced disc degeneration. MRI, particularly when using T1 fat-suppressed sequences, is a valuable diagnostic tool for this condition, helping to distinguish it from other potential causes of T1 hyperintensity. Recognizing fatty degeneration is essential, as failure to do so could lead to confusion with other conditions such as calcification, hemorrhage, or mucin. To accurately confirm fatty degeneration, it is important to incorporate fat-saturated sequences into the imaging protocol alongside routine T1 and T2 sequences. We believe that awareness of this finding will serve as a valuable learning opportunity for clinicians and radiologists, helping to prevent misinterpretation and facilitating a more precise diagnosis.

## Consent

Patient information was de-identified and written informed consent for publication was obtained to publish this case report in accordance with the journal’s patient consent policy.

## Guarantor

Shritik Devkota.
